# Rapid Antigen Processing and Presentation of a Protective and Immunodominant HLA-B*27-restricted Hepatitis C Virus-specific CD8^+^ T-cell Epitope

**DOI:** 10.1371/journal.ppat.1003042

**Published:** 2012-11-29

**Authors:** Julia Schmidt, Astrid K. N. Iversen, Stefan Tenzer, Emma Gostick, David A. Price, Volker Lohmann, Ute Distler, Paul Bowness, Hansjörg Schild, Hubert E. Blum, Paul Klenerman, Christoph Neumann-Haefelin, Robert Thimme

**Affiliations:** 1 Department of Medicine II, University Hospital Freiburg, Freiburg, Germany; 2 Faculty of Biology, University of Freiburg, Freiburg, Germany; 3 Centre of Chronic Immunodeficiency, University of Freiburg, Freiburg, Germany; 4 Nuffield Department of Clinical Neurosciences, Division of Clinical Neurology, Weatherall Institute of Molecular Medicine, Oxford University, Oxford, United Kingdom; 5 Institute of Immunology, University Medical Center of the Johannes Gutenberg University of Mainz, Mainz, Germany; 6 Institute of Infection and Immunity, Cardiff University School of Medicine, Cardiff, United Kingdom; 7 Department of Infectious Diseases, University of Heidelberg, Heidelberg, Germany; 8 Medical Research Council Human Immunology Unit, Weatherall Institute of Molecular Medicine, John Radcliffe Hospital, Oxford, United Kingdom; 9 Nuffield Department of Clinical Medicine, Oxford, United Kingdom; Nationwide Children's Hospital, United States of America

## Abstract

HLA-B*27 exerts protective effects in hepatitis C virus (HCV) and human immunodeficiency virus (HIV) infections. While the immunological and virological features of HLA-B*27-mediated protection are not fully understood, there is growing evidence that the presentation of specific immunodominant HLA-B*27-restricted CD8+ T-cell epitopes contributes to this phenomenon in both infections. Indeed, protection can be linked to single immunodominant CD8+ T-cell epitopes and functional constraints on escape mutations within these epitopes. To better define the immunological mechanisms underlying HLA-B*27-mediated protection in HCV infection, we analyzed the functional avidity, functional profile, antiviral efficacy and naïve precursor frequency of CD8+ T cells targeting the immunodominant HLA-B*27-restricted HCV-specific epitope as well as its antigen processing and presentation. For comparison, HLA-A*02-restricted HCV-specific epitopes were analyzed. The HLA-B*27-restricted CD8+ T-cell epitope was not superior to epitopes restricted by HLA-A*02 when considering the functional avidity, functional profile, antiviral efficacy or naïve precursor frequency. However, the peptide region containing the HLA-B*27-restricted epitope was degraded extremely fast by both the constitutive proteasome and the immunoproteasome. This efficient proteasomal processing that could be blocked by proteasome inhibitors was highly dependent on the hydrophobic regions flanking the epitope and led to rapid and abundant presentation of the epitope on the cell surface of antigen presenting cells. Our data suggest that rapid antigen processing may be a key immunological feature of this protective and immunodominant HLA-B*27-restricted HCV-specific epitope.

## Author Summary

HLA-B*27 has a protective effect in hepatitis C virus (HCV) infection which could be linked to a single highly immunodominant HLA-B*27-restricted CD8+ T-cell epitope. However, the immunological mechanisms determining this protective effect are poorly understood. In this study, we analyzed multiple immunological determinants that may contribute to the protective role of the HLA-B*27-restricted HCV-specific epitope and its strong immunodominance and compared them with HLA-A*02-restricted HCV-specific epitopes. Our data indicate that the protective effect of the HLA-B*27-restricted epitope cannot be explained by a higher sensitivity for antigen stimulation, a higher proportion of effector-functions or a superior ability to inhibit viral replication of epitope-specific CD8+ T cells. We also did not find a higher naïve precursor frequency of HLA-B*27-restricted CD8+ T cells. However, we could show that the peptide region containing the HLA-B*27-restricted epitope is characterized by rapid antigen processing that was mostly due to the hydrophobic flanking regions of the epitope. This results in a faster presentation of the epitope at the cell surface of antigen presenting cells. Our results suggest that rapid antigen processing may be a key mechanism contributing to the protective effect of the immunodominant HLA-B*27-restricted epitope. These findings have clear implications for the design of antiviral vaccines.

## Introduction

The human leukocyte antigen (HLA) B*27 is associated with a high rate of spontaneous viral clearance in hepatitis C virus (HCV) infection [1], [2] and with slow disease progression in human immunodeficiency virus (HIV) infection [3], [4]. In both infections, the protective role has been linked to single immunodominant CD8+ T-cell epitopes [5]–[9]. Virological and immunological mechanisms contribute to the HLA-B*27-mediated protection. For example, extensive virological studies have demonstrated that viral escape from CD8+ T-cell responses that target the protective immunodominant HLA-B*27-restricted epitopes in both HIV and HCV infection is difficult to achieve and requires the accumulation of several mutations. In HIV infection, three mutations within and outside the immunodominant HLA-B*27-restricted epitope (KK10) are required for viral escape: the first mutation has an immunmodulatory effect, a second mutation compensates for viral fitness costs, and a third mutation abrogates HLA binding [10]–[13]. In HCV infection, viral escape mutations are not tolerated at the HLA-B*27 binding anchors of the epitope due to a major impact on viral replicative fitness. Mutations at T-cell-receptor contact residues can occur in this otherwise highly conserved region; however, several of these mutations are required for full escape due to broad T-cell cross-recognition of viral variants [14]. Thus, in both infections, related but distinct mechanisms constrain virological escape and contribute to protection.

In contrast, the immunological mechanisms that contribute to HLA-B*27-mediated protection are less well understood. It is possible that certain characteristics of HLA-B*27-restricted CD8+ T cells may contribute to the protective effect. Indeed, functional avidity, defined as the sensitivity of CD8+ T cells to antigenic stimulation, has been proposed to correlate with the outcome of viral infection [15]–[19]. For example, one study found that the functional avidity of HLA-B*27-restricted HIV-specific CD8+ T-cell responses directed against the protective immunodominant KK10 epitope was higher in comparison to responses directed against epitopes restricted by other HLA-alleles [15]. Others reported that the functional avidity of KK10-specific CD8+ T-cell responses was comparable to that of responses targeting subdominant HLA-B*27-restricted epitopes derived from HIV [18]. It has also been suggested that immunodominant HLA-B*27-restricted HIV-specific CD8+ T-cell responses display more potent antiviral efficacy compared to responses targeting subdominant HLA-B*27-restricted epitopes [18].

Another factor that has been suggested to contribute to the superior control of HIV replication is the polyfunctionality of the immunodominant KK10-specific CD8+ T cells [15]. Polyfunctionality is characterized by the simultaneous production of multiple effector-molecules such as CD107a, IFN-γ, IL-2, MIP-1β and TNF-α.

However, since HLA-B*27-mediated protection is also closely linked to the immunodominance of HLA-B*27-restricted virus-specific CD8+ T-cell responses, it is tempting to speculate that factors determining immunodominance may help explain this protective effect. Immunodominance is expected to be determined early after infection when naïve antigen-specific CD8+ T cells encounter their antigen and the first responses start to emerge. Factors that contribute to CD8+ T-cell immunodominance include antigen processing and presentation, abundance of peptide-major histocompatibility complex class I (pMHCI) molecules on antigen-presenting cells (APCs) and the number of naïve T cells that express complementary T-cell receptors [20]. Studies primarily performed in mouse models have shown that the immunodominance hierarchy and the magnitude of T-cell responses are shaped by the number of antigen-specific naïve CD8+ T cells [21]–[23]. Consistent with these findings, we have shown that the immunodominance hierarchy of HLA-A*02-restricted HCV-specific CD8+ T-cell responses observed in chronically HCV infected subjects is related to the frequency of naïve CD8+ precursors [24]. However, there is also growing evidence that immunodominance is largely influenced by differences in antigen processing [25]–[28]. One study has demonstrated that antigen processing strongly influences CD8+ T-cell response hierarchies in HIV infection as the amount of epitope produced correlated with CD8+ T-cell response magnitude and frequency [29]. Moreover, proteasomal cleavage of HIV Gag-derived polypeptides by the immunoproteasome efficiently generated precursors of the HIV-specific HLA-B*27-restricted protective immunodominant KK10 epitope as well as minute amounts of the optimal epitope itself [29], [30]. The ‘optimal’ epitope is defined as the epitope form that results in the greatest *in vitro* stimulation of epitope-specific CD8+ T-cell responses.

In this comprehensive study, we set out to elucidate the immunological factors that mediate the protection afforded by HLA-B*27 in HCV infection. Compared to HLA-A*02-restricted HCV-specific CD8+ T-cell responses, we found that the protective effect of the immunodominant HLA-B*27-restricted HCV-specific epitope is not clearly linked to differences in the intrinsic properties of cognate CD8+ T-cell populations, but rather to more rapid and efficient antigen processing and presentation. These results suggest that both immunodominance and protection in this case are associated with the kinetics and efficacy of epitope generation.

## Results

### Functional avidity of protective immunodominant HLA-B*27-restricted HCV-specific CD8+ T-cell populations

In a first set of experiments, we assessed the functional avidity of HCV-specific CD8+ T-cell populations from chronically HCV infected subjects. For comparative purposes, CD8+ T-cell populations specific for three frequently recognized HLA-A*02-restricted epitopes were evaluated in addition to those specific for the immunodominant HLA-B*27-restricted NS5B_2841_ epitope (**Table 1**). All four epitopes bind comparably and with high affinity to their restricting HLA class I molecule [31]. In HLA-A*02+ tested subjects only one or a maximum of two epitope-specific CD8+ T cell responses were detectable and thus analyzed. It is important to note, that in all functional assays performed in this study, short term expanded HCV-specific CD8+ T-cell lines (14 days) were used. Circulating HCV-specific CD8+ T cells in chronically infected subjects are only present at a very low frequency (usually <0.1% of total CD8+ T cells); this does not allow the performance of functional analyses.

**Table 1:** HLA-A*02 and -B*27 restricted epitopes.

Allele	Amino Acid Position	Peptide Length	Sequence	IC50 (nM)*
HLA-A*02	NS3_1073_	9	CINGVCWTV	66.5
HLA-A*02	NS3_1406_	10	KLVALGINAV	52.8
HLA-A*02	NS5B_2594_	9	ALYDVVSKL	11.8
HLA-B*27	NS5B_2841_	9	ARMILMTHF	28.4

* IC50 was determined by using the immune epitope database (IEDB, http://www.immuneepitope.org/).

**Figure 1:**
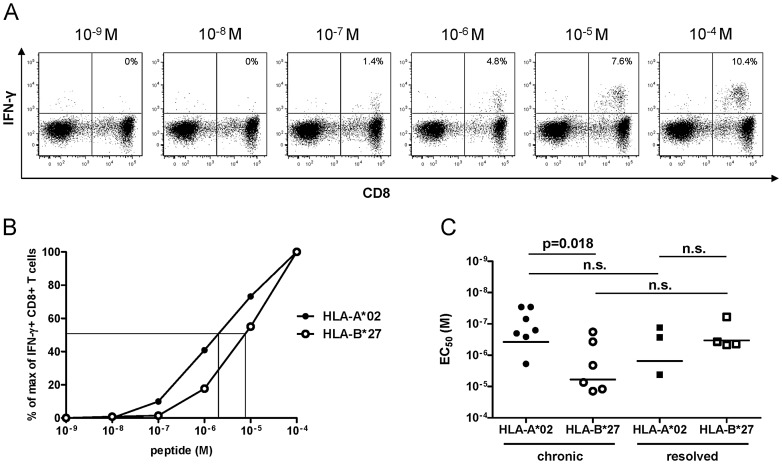
Functional avidity of HCV-specific CD8+ T cells. (A) Representative flow cytometry plots showing intracellular IFN-γ staining of a CTL line exposed to target cells pulsed with a range of peptide concentrations. PBMCs from a subject chronically infected with HCV were stimulated for two weeks with the HLA-A*02-restricted NS3_1073_ epitope. Prior to intracellular IFN-γ staining, the CTL line was cocultured for 5 hours with HLA-A*02+ EBV-immortalized B-cell lines (B-LCLs) pulsed with serially diluted concentrations of the cognate peptide. The background value from the negative control (B-LCLs without peptide loading) was subtracted from all measured response frequencies (IFN-γ+ CD8+/total CD8+). (B) Representative data from intracellular IFN-γ staining of a CTL line specific for the HLA-A*02-restricted NS3_1073_ epitope (filled circles) and a CTL line specific for the HLA-B*27-restricted NS5B_2841_ epitope (open circles) across a range of peptide concentrations. The background value from the negative control (B-LCLs without peptide loading) was subtracted from all measured response frequencies (IFN-γ+ CD8+/total CD8+), which were then normalized to the maximum response by defining the smallest value as 0% and the largest value as 100%. (C) The functional avidity of CTL lines, derived from chronically HCV infected subjects, specific for HLA-A*02-restricted epitopes (n = 7, filled circles) and the immunodominant HLA-B*27-restricted epitope NS5B_2841_ (n = 6, open circles), as well as of CTL lines derived from subjects with resolved infection specific for HLA-A*02-restricted epitopes (n = 3, filled squares) and the immunodominant HLA-B*27-restricted epitope NS5B_2841_ (n = 4, open squares). Functional avidity was determined as the concentration of peptide required to achieve half-maximal IFN-γ induction (EC_50_). P-values were calculated using the Mann-Whitney U-test. Horizontal bars represent mean values.

After two weeks of peptide-specific expansion, cytotoxic T lymphocyte (CTL) lines were stimulated with HLA-B*27+ or HLA-A*02+ subject-derived EBV-immortalized B-cell lines pulsed with serial peptide concentrations (**Figure 1A**); the response EC_50_ was quantified as the exogenous peptide concentration required to yield half-maximal frequencies of cells producing intracellular IFN-γ, as shown in **Figure 1B**. Importantly, HLA-B*27-restricted CD8+ T cells displayed a significantly lower mean functional avidity (indicated by a significantly higher mean EC_50_ value) compared to HLA-A*02-restricted CD8+ T cells from the same subject group (**Figure 1C**). Since the presence of chronic infection may affect the properties of virus-specific CD8+ T-cell populations, we also analyzed the functional avidity of CD8+ T cells obtained from subjects with resolved HCV infection. Interestingly, HLA-B*27-restricted CD8+ T cells from subjects with resolved HCV infection displayed a higher mean functional avidity compared to cells obtained from chronically infected HLA-B*27+ subjects although this difference was not statistically significant (**Figure 1C**). However, this did not exceed the mean functional avidity observed for HLA-A*02-restricted epitopes targeted in chronic or resolved infection. Thus, superior functional avidity is not a characteristic of HLA-B*27-restricted CD8+ T cells specific for the protective immunodominant NS5B_2841_ epitope when compared to HLA-A*02-restricted CD8+ T-cell populations.

### Functional profile of protective immunodominant HLA-B*27-restricted HCV-specific CD8+ T-cell populations

**Figure 2:**
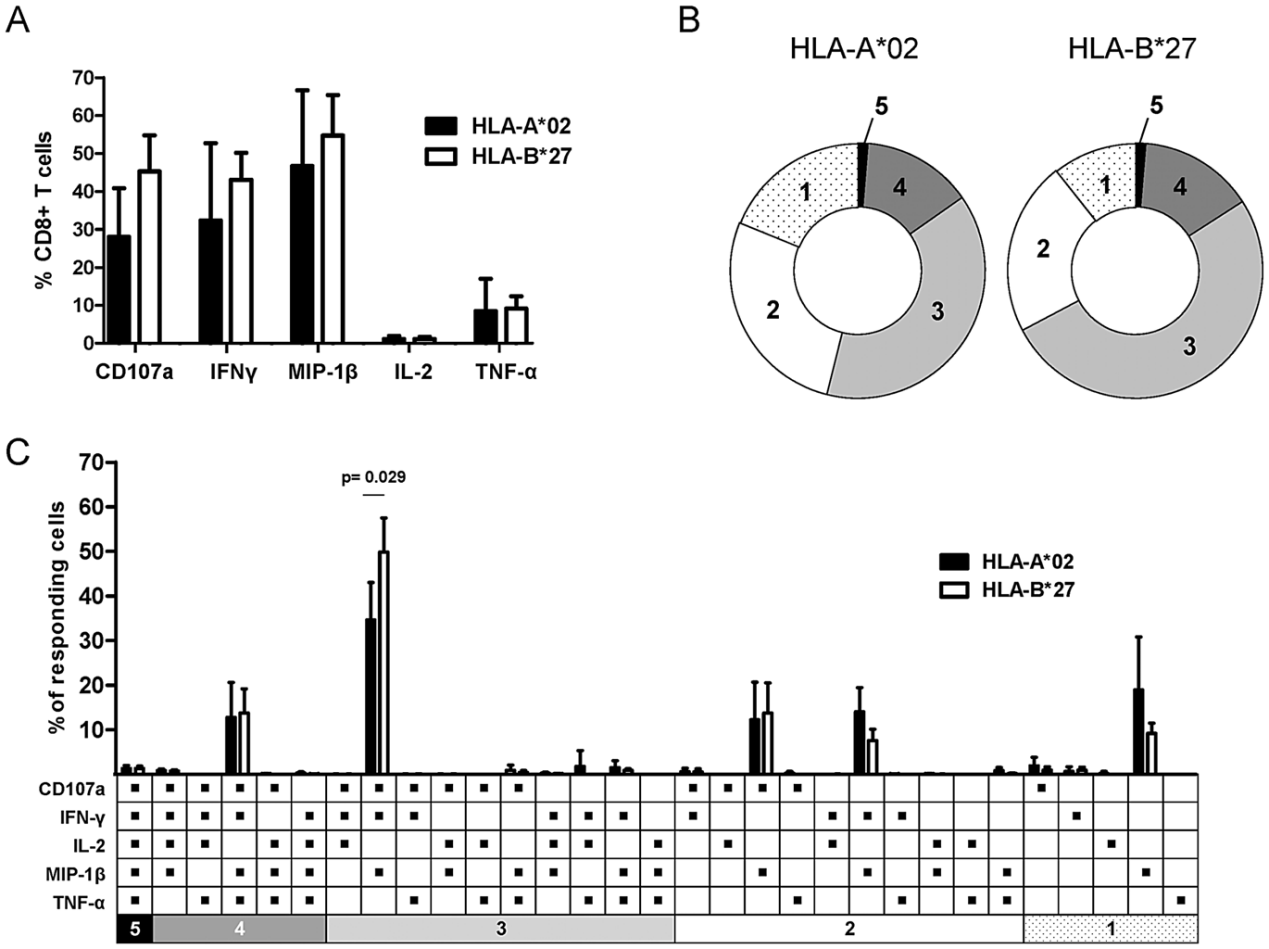
Functional profile of HCV-specific CD8+ T cells. (A) Cytokine production and CD107a mobilization of CTL lines, derived from chronically HCV infected subjects specific for HLA-A*02-restricted epitopes (n = 4, black bars) and the immunodominant HLA-B*27-restricted epitope NS5B_2841_ (n = 4, white bars). Prior to intracellular multi-cytokine staining CTL lines were stimulated for 5 hours with specific peptides. The background value from the negative control (without peptide) was subtracted from all measured response frequencies (cytokine+ CD8+/total CD8+). Data are presented as mean ± SD. (B) Pie charts showing the mean multifunctionality of HLA-A*02-restricted (n = 4) and HLA-B*27-restricted (n = 4) HCV-specific CD8+ T-cell lines (one to five functions: CD107a, IFN-γ, IL-2, MIP-1β and TNF-α). Responding cells are grouped by the number of functions indicated by the numbers in the pie charts and matched to the color code in figure 2C. (C) Detailed functional profile of HLA-A*02-restricted (n = 4, black bars) and HLA- B*27-restricted (n = 4, white bars) HCV-specific CD8+ T-cell lines. Bars represent frequency of CD8+ T cells displaying the indicated combination of functions within the total population of responding CD8+ T cells. P-values were calculated using the Mann-Whitney U-test. Data are presented as mean ± SD.

In order to compare the functional profile of HLA-B*27- and HLA-A*02-restricted CD8+ T cells from chronically HCV-infected subjects we performed intracellular multi-cytokine staining. Upon peptide-specific stimulation, HLA-B*27- and HLA-A*02-restricted CTL lines produced the same array of cytokines with even similar quantities (**Figure 2A**). Particularly, CD107a was mobilized and IFN-γ, MIP-1β and TNF-α were produced by CD8+ T cells restricted by both HLA-alleles. However, production of IL-2 by all virus-specific CD8+ T cells was limited (**Figure 2A**) and neither HLA-B*27- nor HLA-A*02-restricted CD8+ T cells produced IL-4, IL-10, IL-17A or IL-22 (data not shown). The direct number of functions (polyfunctional profile) of individual CD8+ T cells is shown in **Figure 2B and 2C**. Although CD8+ T cells restricted by both alleles produced similar amounts of the same cytokines, individual HLA-B*27-restricted CD8+ T cells produced more cytokines simultaneously compared to HLA-A*02-restricted CD8+ T cells. Importantly, they showed a higher proportion of CD107a+, IFN-γ+ and MIP-1β+ cells. These results suggest that HLA-B*27- and HLA-A*02-restricted CD8+ T cells derived from chronically infected subjects have similar functional qualities with comparable quantities even though their polyfunctional profile at a cell based level may slightly differ.

### Antiviral efficacy of protective immunodominant HLA-B*27-restricted HCV-specific CD8+ T-cell populations

**Figure 3:**
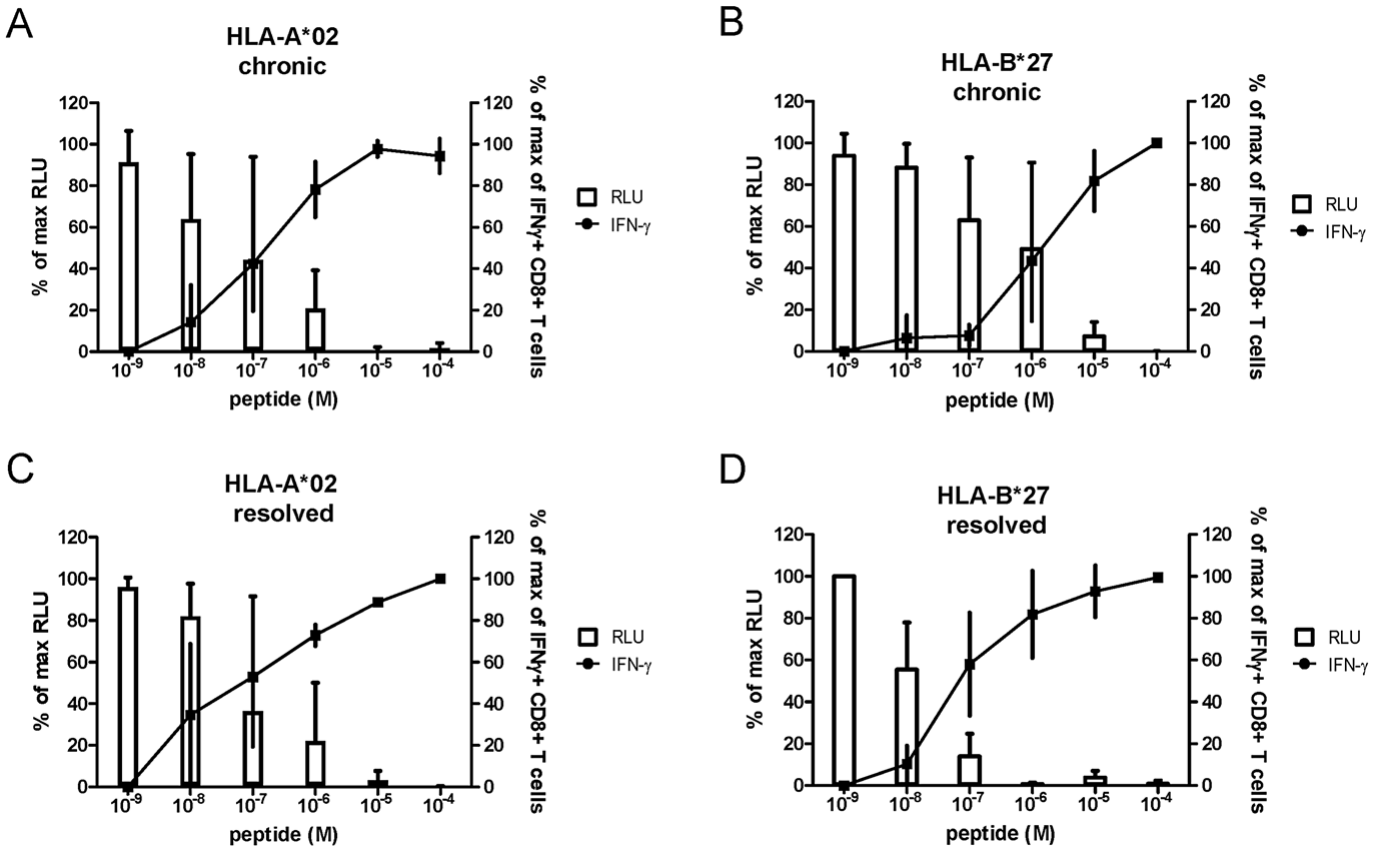
Antiviral efficacy of HCV-specific CD8+ T cells. Antiviral efficacy (white bars) and functional avidity (filled circles) of CTL lines, derived from subjects with a chronic HCV infection specific for (A) HLA-A*02-restricted epitopes (n = 3) and (B) the immunodominant HLA-B*27-restricted epitope NS5B_2841_ (n = 3) as well as of CTL lines derived from subjects with resolved infection specific for (C) HLA-A*02-restricted epitopes (n = 3) and (D) the immunodominant HLA-B*27-restricted epitope NS5B_2841_ (n = 3). HuH7_A2_HCV and HuH7_B27_HCV cell lines were pulsed with a range of cognate peptide concentrations and cocultured for 24 hours with CTL lines at an E∶T ratio of 1∶1. Inhibition of viral replication was measured by luciferase activity. As a reference, intracellular IFN-γ staining at corresponding peptide concentrations is indicated. Measured RLUs and IFN-γ responses were normalized to maximum viral replication or maximum IFN-γ response, respectively, by defining the smallest value as 0% and the largest value as 100%. Data are presented as mean ± SD.

Next, we analyzed whether HLA-B*27-restricted NS5B_2841_ epitope-specific CD8+ T cells exert superior antiviral efficacy compared to CD8+ T cells of other specificities. In HIV infection, it has been suggested that a protective immunodominant HLA-B*27-restricted CD8+ T-cell response is characterized by superior antiviral activity [18]. To address this possibility, we used human hepatoma HuH7 cells harboring a JFH1-based selectable subgenomic luciferase replicon to measure the inhibition of HCV replication by CD8+ T cells. These replicon cells were transduced with lentiviral vectors expressing HLA-A*02 or HLA-B*27 with a selectable marker conferring blasticidin resistance controlled by the constitutive cellular EF1a promoter, as described previously [32]. The resulting cell lines showed strong and homogeneous expression of HLA-A*02 or HLA-B*27, respectively, by flow cytometric analysis (data not shown). The inhibitory effect on HCV replication was measured by determining luciferase activity, which is fully dependent on HCV RNA replication and correlates precisely with intracellular levels of viral RNA antigens [32]. Since the subgenomic replicon is based on the genotype 2a isolate JFH1 and its sequence is not cross-recognized by the respective CD8+ T cells specific for genotype 1a peptides, pulsing with respective peptide is required (genotype 1a sequence, see **Table 1**). HuH7 cells expressing HLA-A*02 were pulsed with increasing concentrations of specific HLA-A*02-restricted peptides and HuH7 cells expressing HLA-B*27 were pulsed with increasing concentrations of the dominant HLA-B*27 peptide. The target cells were then cocultured with respective peptide-specific CTL lines at an effector-to-target (E∶T) ratio of 1∶1. Importantly, as shown in **Figure 3**, virus-specific CD8+ T cells blocked HCV replication in a peptide dose-dependent manner that correlated precisely with the amount of IFN-γ secreted by the virus-specific T cells. Of note, HLA-B*27-restricted CD8+ T cells specific for the NS5B_2841_ epitope derived from chronically infected subjects did not mediate superior antiviral efficacy. HLA-A*02-restricted CD8+ T cells started to inhibit HCV replication at a lower peptide concentration (10^−8^) compared to HLA-B*27-restricted CD8+ T cells (**Figure 3A and B**). We additionally analyzed the antiviral efficacy of CD8+ T cells derived from subjects with resolved HCV infection (**Figure 3C and D**). HLA-B*27-restricted CD8+ T cells from subjects with resolved HCV infection displayed a higher antiviral efficacy compared to cells obtained from chronically infected HLA-B*27+ subjects. However, they were not much superior to HLA-A*02-restricted CD8+ T cells obtained from subjects with chronic or resolved infection. These findings show that the antiviral efficacy is correlated with the functional avidity of CD8+ T cells and suggest that the protective effect of the immunodominant HLA-B*27-restricted epitope is not explained by the antiviral efficacy of epitope-specific CD8+ T cells.

### Naïve precursor frequency of protective immunodominant HLA-B*27-restricted HCV-specific CD8+ T cells

**Figure 4:**
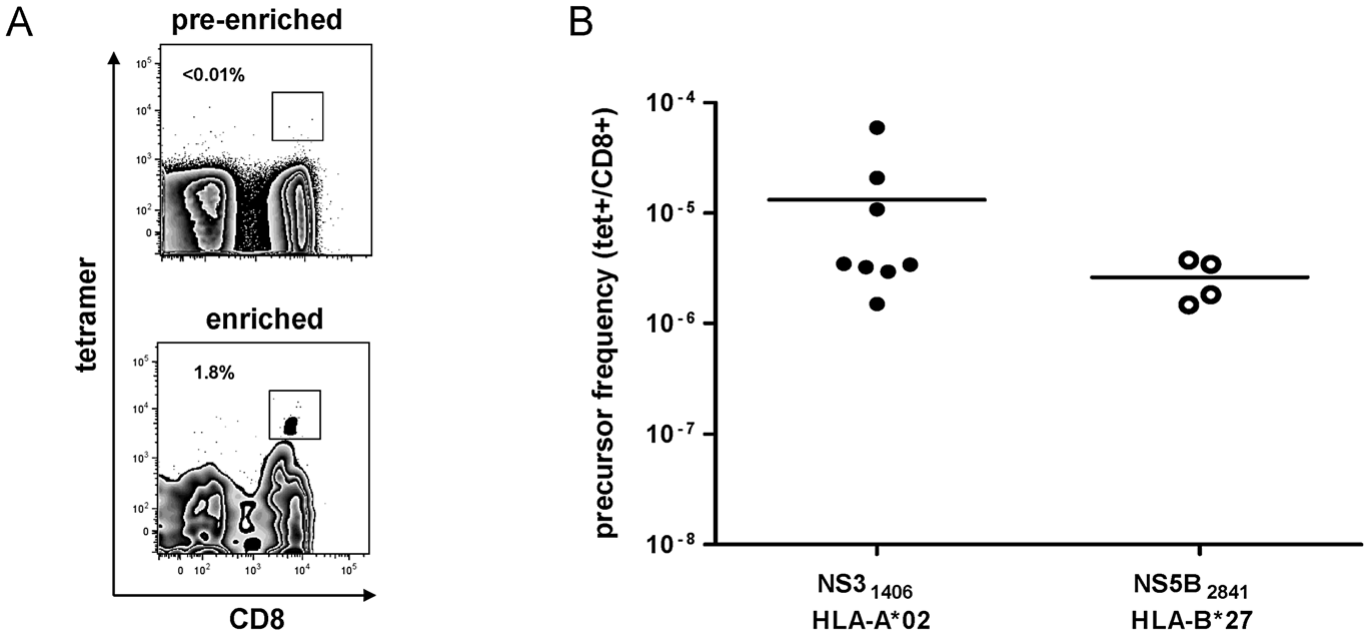
Precursor frequency of HCV-specific CD8+ T cells. (A) Representative flow cytometry plots of PBMCs from a healthy donor displaying staining with the NS5B_2841_/HLA-B*27 tetramer before and after magnetic-bead enrichment. Percentage of tetramer+ CD8+ T cells is indicated. (B) Precursor frequencies of CD8+ T cells specific for NS3_1406_ (HLA-A*02 tetramer, filled circles) and NS5B_2841_ (HLA-B*27 tetramer, open circles) detected in healthy donors after tetramer staining, magnetic-bead enrichment and multiparametric flow cytometric analysis. The enriched tetramer+ CD8+ T-cell populations were stained for CD45RA, CD27, CCR7 and CD11a. The number of phenotypically naïve (CD45RA^hi^, CD27^hi^, CCR7^hi^, CD11a^low^) tetramer+ CD8+ T cells relative to the number of total CD8+ T cells is indicated. Horizontal bars represent mean values.

Next, we asked whether the strong immunodominance of the NS5B_2841_ epitope might be dictated by a high frequency of naïve CD8+ T-cell precursors. To address this issue, we analyzed naïve precursor frequencies of CD8+ T cells specific for the NS5B_2841_ epitope and compared it to the well described immunodominant HLA-A*02-restricted NS3_1406_ epitope. Of note, we recently showed that the immunodominance of this latter epitope is linked to a relatively high frequency of epitope-specific naïve precursors [24]. By using a previously described combination of tetramer staining, magnetic-bead enrichment and multiparametric flow cytometry [33], we were able to detect naïve HCV-specific CD8+ T cells in all four HLA-B*27 and all eight HLA-A*02 healthy donors analyzed (**Figure 4**). Although the cells were not detectable before the enrichment step, they could be clearly identified after enrichment, as shown for one representative subject in **Figure 4A**. The cells displayed a naïve phenotype characterized by high expression of CD45RA, CD27 and CCR7, and low expression of CD11a (data not shown). Of note, the naïve precursor frequency of HLA-B*27-restricted CD8+ T cells specific for NS5B_2841_ was not significantly higher compared to the frequency of the immunodominant HLA-A*02-restricted naïve precursors (**Figure 4B**, data of HLA-A*02-restricted naïve precursors were previously published [24]). These data suggest that the strong immunodominance of the HLA-B*27-restricted NS5B_2841_ epitope is not due to an intrinsically high naïve precursor frequency, at least across HLA restriction elements.

### Proteasomal processing of the protective immunodominant HLA-B*27-restricted NS5B_2841_ epitope

**Figure 5:**
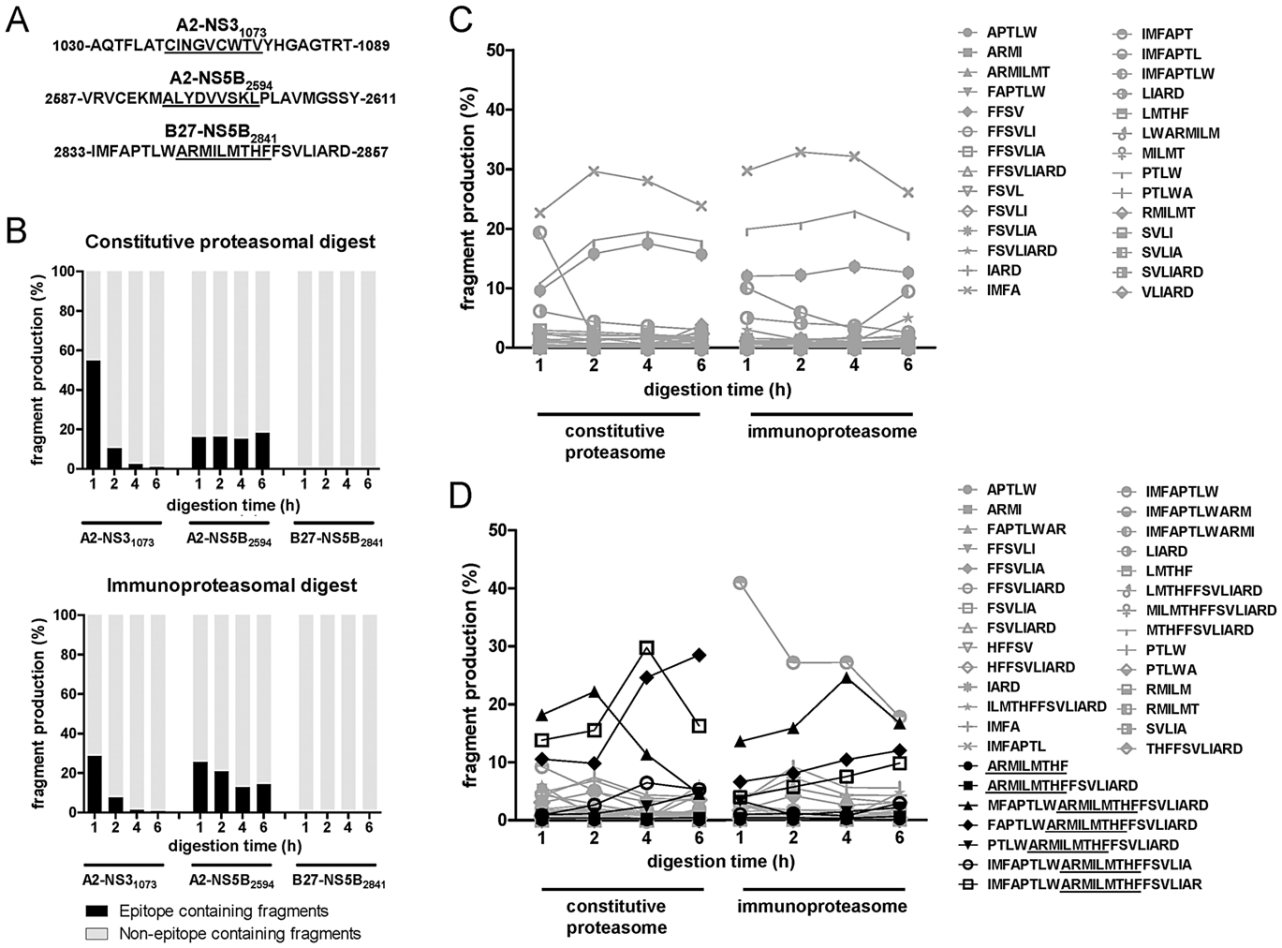
Proteasomal digestion of HCV-specific epitope-containing regions. (A) Peptides of 24 (A2-NS3_1073_) and 25 (A2-NS5B_2594_ and B27-NS5B_2841_) amino acids in length spanning the regions containing the respective epitopes were used for incubation with constitutive proteasomes and immunoproteasomes. Epitope sequences are underlined. (B) Production of epitope-containing and non-epitope-containing cleavage products of the 24-mer (A2-NS3_1073_) and two 25-mer (A2-NS5B_2594_ and B27-NS5B_2841_) peptides incubated with constitutive proteasomes and immunoproteasomes for 1 to 6 hours using standard conditions. The sum of all fragment intensities was set at 100%. Data are representative of triplicate experiments. (C) The relative production of all cleavage products of the B27-NS5B_2841_ peptide by constitutive proteasomes and immunoproteasomes for 1 to 6 hours using standard conditions. All fragments were between 4 and 9 amino acids long. Data are representative of triplicate experiments. (D) The relative production of all cleavage products of the B27-NS5B_2841_ peptide by constitutive proteasomes and immunoproteasomes for 1 to 6 hours using a 4× dilution of both of the proteasomal forms. The abundance of B27-NS5B_2841_-containing fragments ending in a C-terminal ^2857^D represents a maximum as this corresponds to the end of the 25-mer peptide substrate. All fragments containing the NS5B_2841_ epitope are highlighted using black symbols. The NS5B_2841_ epitope sequence is underlined. Data are representative of triplicate experiments.

**Figure 6:**
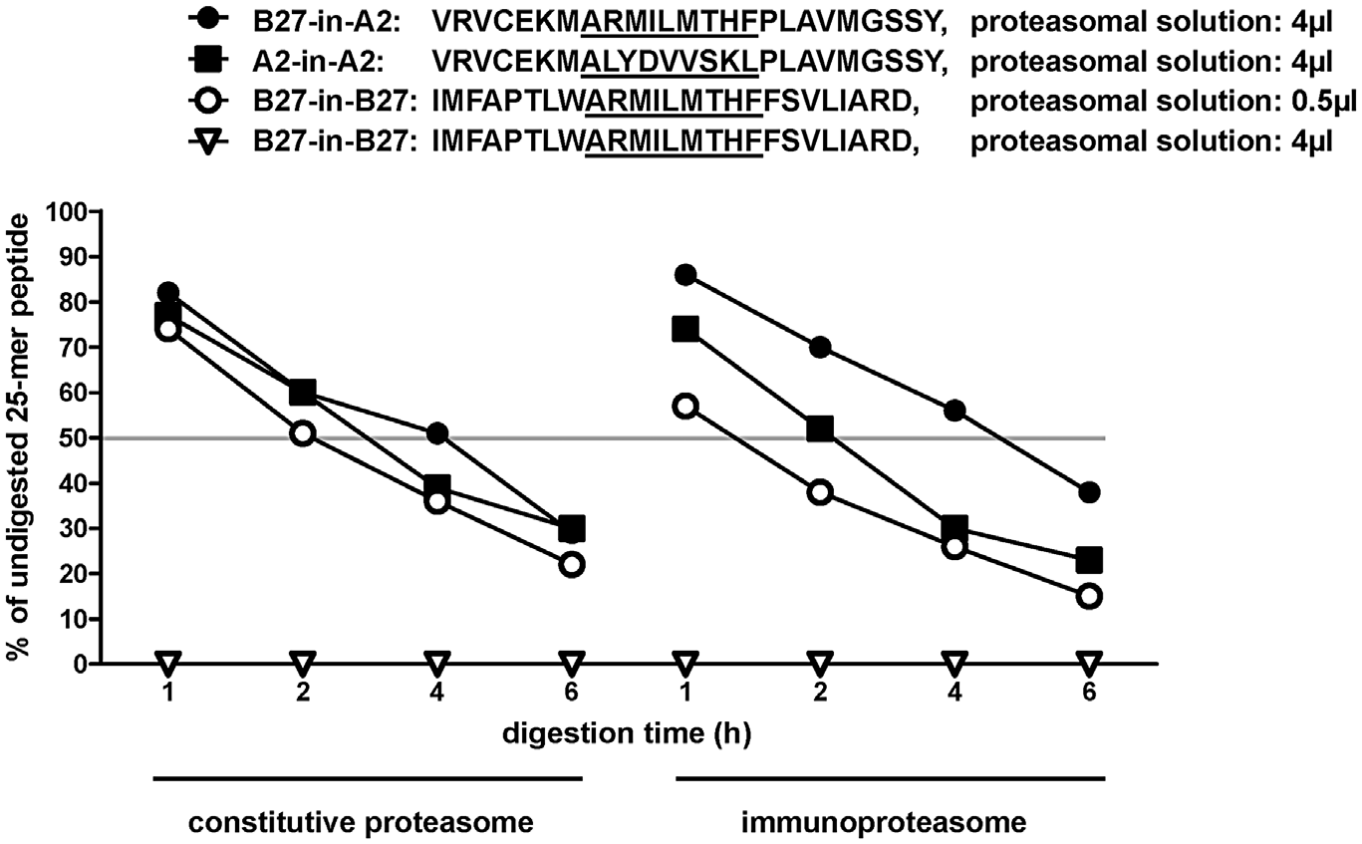
Epitope precursor degradation rates of natural HLA-A*02- and HLA-B*27-restricted epitopes as well as of an artificial chimeric epitope. The percentage of undigested 25-mer precursor peptide left following 1, 2, 4, and 6 hours of constitutive and immunoproteasomal digestion, respectively. The amino acid sequences of the examined 25-mer peptides are shown with imbedded optimal epitopes underlined and the amount of purified proteasomal solution is indicated. B27-in-A2 signifies the B27-NS5B_2841_ epitope surrounded by the A2-NS5B_2594_ flanking regions, while A2-in-A2 and B27-in-B27 show these epitopes in their natural sequence contexts. The grey line indicates 50% degradation of the precursor peptide. Data are representative of triplicate experiments.

Recently, it has been shown that antigen processing influences HIV-specific CD8+ T-cell response hierarchies [29]. In case of an HLA-B*27-restricted HIV Gag-derived epitope, immunodominance correlated with abundant proteasomal production of a range of short and long, naturally processed peptide forms containing the optimal epitope [29]. We therefore analyzed whether proteasomal cleavage of the dominant NS5B_2841_ epitope has an influence on the immunodominant response to this epitope. Specifically, we performed a proteasomal digest of a 25-amino acid peptide containing the HLA-B*27-restricted dominant NS5B_2841_ epitope, as well as two peptides containing the HLA-A*02-restricted epitopes NS3_1073_ and NS5B_2594_ (**Figure 5A**), using constitutive proteasomes and immunoproteasomes in parallel over a time course of six hours. Subsequently, the resulting fragments were analyzed by mass spectrometry. Our initial proteasomal digestion experiments resulted in production of the optimal HLA-A*02-restricted NS3_1073_ and NS5B_2594_ (A2-NS3_1073_ and A2-NS5B_2594_) epitopes, but surprisingly failed to yield any of the HLA-B*27-restricted NS5B_2841_ (B27-NS5B_2841_) epitope or even B27-NS5B_2841_ epitope-containing fragments (**Figure 5B**). Indeed, the 25-mer B27-NS5B_2841_ polypeptide was completely degraded and only peptides of four to nine amino acids in length could be detected (**Figure 5C**). We therefore performed additional digestion experiments with this polypeptide substrate at a four-fold dilution of both proteasomal forms. By using this more sensitive approach, we were able to detect small amounts of the optimal B27-NS5B_2841_ epitope after four to six hours of immunoproteasomal digestion. Importantly, we also observed six additional longer peptides containing the B27-NS5B_2841_ epitope, each of which was present at concentrations exceeding that of the optimal B27-NS5B_2841_ epitope by up to 300-fold (**Figure 5D**; the optimal B27-NS5B_2841_ epitope as well as all B27-NS5B_2841_ epitope-containing fragments are highlighted in black). Because some of these fragments ended at the C-terminal end of the analyzed 25-mer peptide, and production thus was a maximum estimate, we performed additional proteasomal digestions of an overlapping, but more C-terminal 25-mer peptide encompassing B27-NS5B_2841_. These proteasomal digestions demonstrated cleavage C-terminal of the first analyzed fragment (**Figure S1**). Although epitope production was less than indicated by the maximum estimates of our first experiment, the overall production of the B27-NS5B_2841_ epitope-containing fragments still comprised between 8–32% of all digestion products (data not shown). To investigate whether the rapid proteasomal processing was due to the B27-NS5B_2841_ sequence itself, or to the very hydrophobic regions flanking this epitope, we generated an artificial chimeric 25-mer peptide containing the B27-NS5B_2841_ epitope imbedded in the regions flanking the A2-NS5B_2594_ epitope (termed B27-in-A2). We found that this B27-in-A2 peptide degraded in a manner more similar to the original A2-in-A2 25-mer peptide than the original B27-in-B27 peptide (**Figure 6**). Indeed, the chimeric B27-in-A2 peptide degraded slightly more slowly than the A2-in-A2 peptide following immunoproteasomal digestion and although we doubled the amount of proteasome used in this experiment, we could not increase the rate of degradation to that of the original B27-in-B27 peptide. Consequently, the hydrophobicity of the B27-NS5B_2841_ epitope flanking region is critical to the rapid processing of this epitope. Our data furthermore suggests that the combination of epitope and flanking region affect the overall peptide degradation rate.

In order to analyze whether during degradation of the chimeric B27-in-A2 peptide the optimal epitope is generated, we compared the amount of optimal 9-mer B27-NS5B_2841_ epitope generated following digestion of the artificial chimeric B27-in-A2 peptide with that made following processing of the natural B27-in-B27 peptide (**Figure S2**). Our data show that more of the optimal B27-NS5B_2841_ epitope was released following both constitutive and immunoproteasomal digestion when the epitope was in the A2-NS5B_2594_ flanking context, although the epitope was produced more slowly. It is important to note, that we compared only the amounts of optimal 9-mer epitope. Longer versions of the B27-NS5_2841_ epitope are not produced following processing of the artificial chimeric B27-in-A2 construct while these are made in great amounts when the 25-mer peptide with the natural B27-in-B27 amino acid sequence is processed.

**Figure 7:**
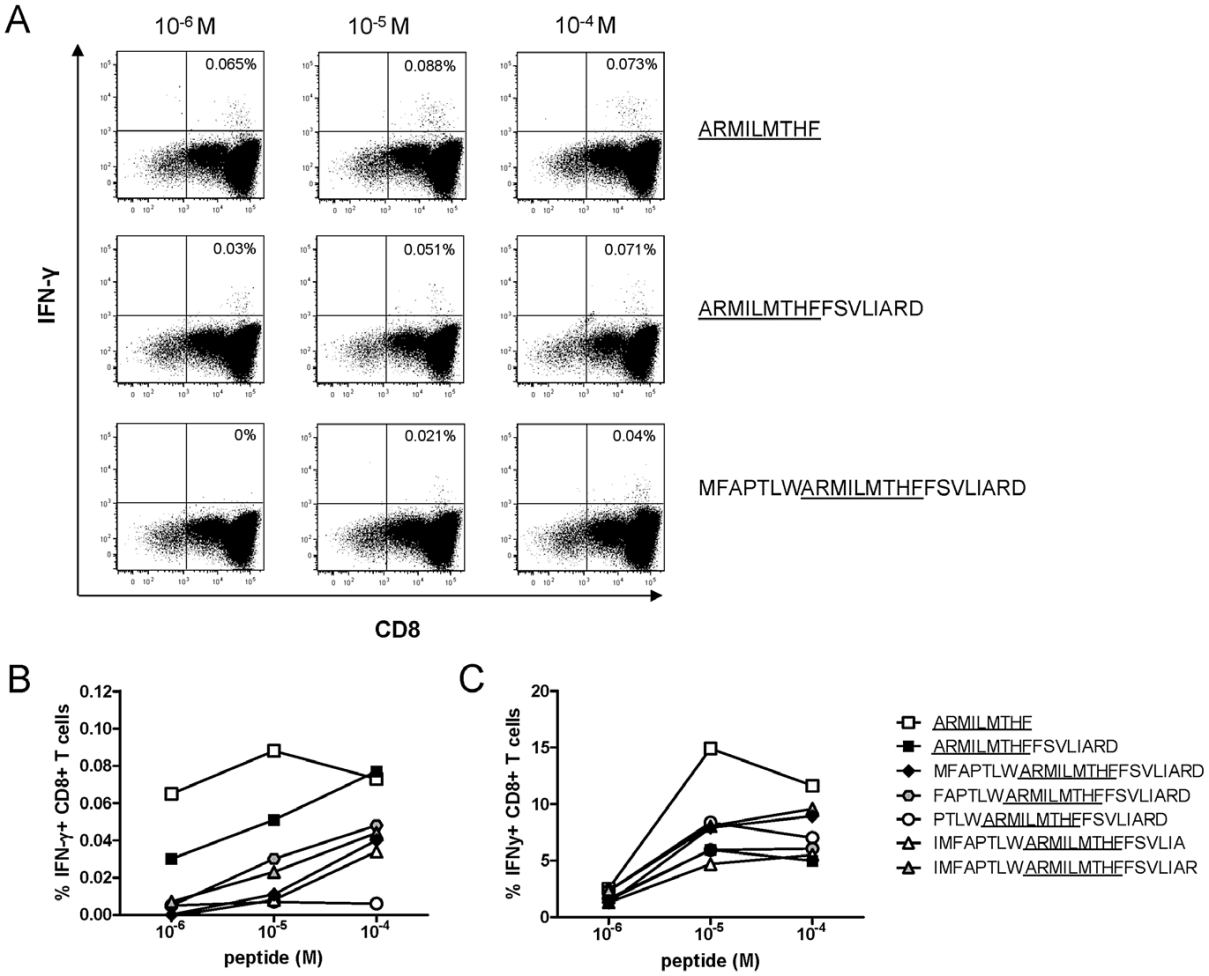
Cross-recognition patterns of all B27-NS5B_2841_-containing fragments derived from immunoproteasomal digestion. (A) Representative flow cytometry plots showing intracellular IFN-γ staining with CD8+ T cells from one subject across a range of peptide concentrations. PBMCs from HLA-B*27+ subjects chronically infected with HCV were enriched for CD8+ T cells and expanded non-specifically for 14 days. The background value from the negative control (without peptide loading) was subtracted from all measured response frequencies (IFN-γ+ CD8+/total CD8+). Tested peptide sequences are indicated. (B) Representative cross-recognition patterns of all naturally processed B27-NS5B_2841_-containing fragments by enriched and non-specifically expanded CD8+ T cells (see Figure 7A). Responses were measured by intracellular IFN-γ staining against serial dilutions of all 7 B27-NS5B_2841_-containing peptide forms. The background value from the negative control (without peptide loading) was subtracted from all measured response frequencies (IFN-γ+ CD8+/total CD8+). Symbol code for peptide sequences is indicated in Figure 7C. (C) Representative cross-recognition patterns of the NS5B_2841_- peptide by PBMCs specifically expanded by all naturally processed B27-NS5B_2841_-containing fragments. PBMCs from subjects chronically infected with HCV were stimulated for two weeks with all 7 B27-NS5B_2841_-containing peptide forms separately (see indicated symbol code). Responses were measured by intracellular IFN-γ staining against serial dilutions of the NS5B_2841_- peptide. The background value from the negative control (without peptide loading) was subtracted from all measured response frequencies (IFN-γ+ CD8+/total CD8+).

As it has been shown previously that HLA-B*27 is able to bind and present peptides that are N- or C-terminally extended fragments of an optimal epitope [29], [34], we next examined the cross-recognition of the seven B27-NS5B_2841_ epitope-containing peptide forms using non-specifically expanded CD8+ selected T cells derived from two chronically HCV infected HLA-B*27+ subjects by intracellular cytokine staining for IFN-γ. Importantly, these CD8+ T cells responded to almost all naturally processed B27-NS5B_2841_ epitope-containing peptides (**Figure 7A and B**). In one subject, some of the long peptide variants induced even slightly stronger IFN-γ production compared to the optimal B27-NS5B_2841_ epitope (data not shown). In these experiments, we used non-specifically expanded CD8+ T cells rather than peptide-stimulated T-cell lines because they best represent the range of CD8+ T-cell populations found in subjects *in vivo*. To confirm specificity, we also analyzed the capability of the NS5B_2841_ epitope-containing peptide forms to stimulate the expansion of epitope-specific CD8+ T cells derived from chronically infected HLA-B*27+ subjects; all tested peptide forms were able to stimulate epitope-specific CD8+ T-cell expansions in culture (**Figure 7C**).

### Rapid and efficient presentation of the protective immunodominant HLA-B*27-restricted HCV-specific epitope

**Figure 8:**
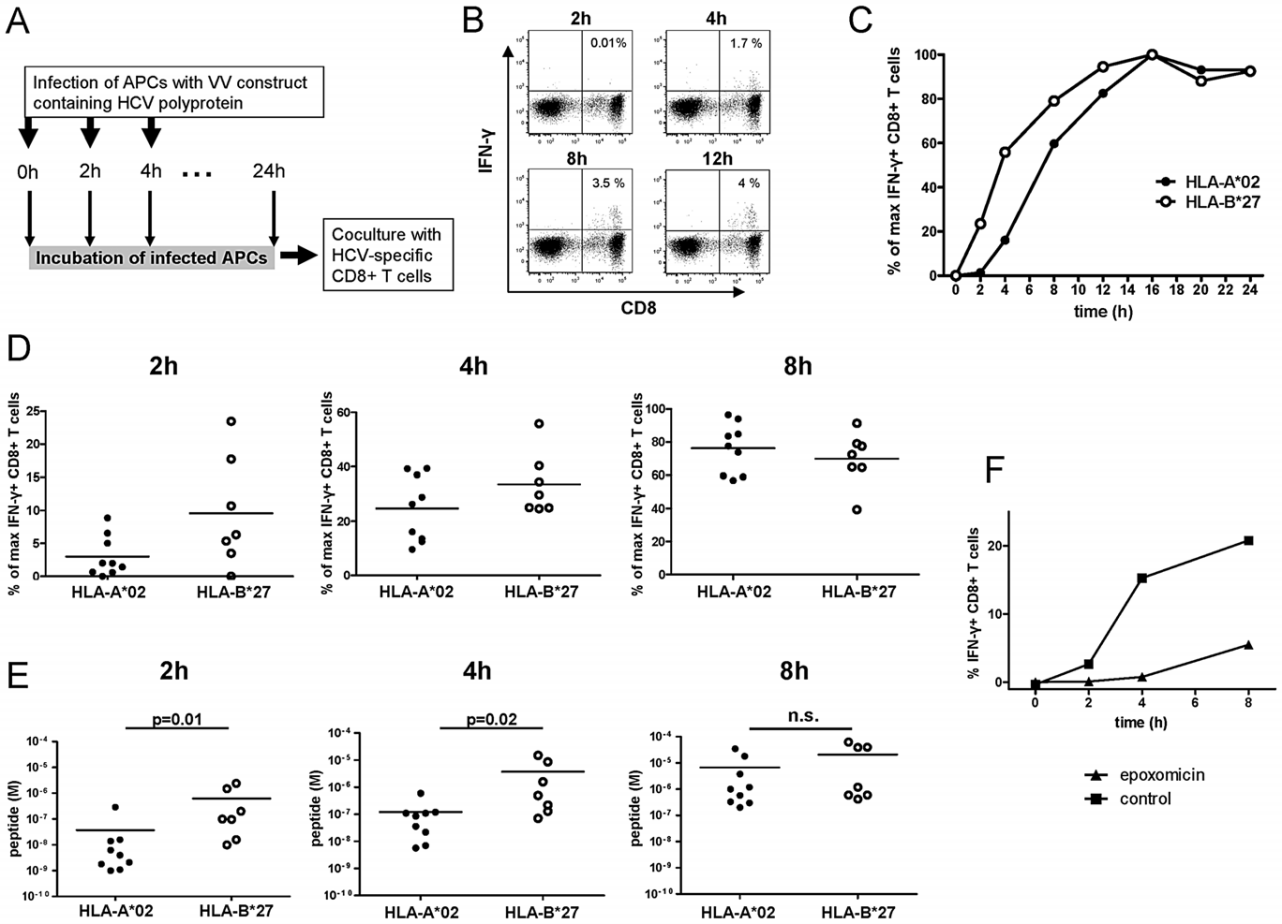
Processing and presentation of HCV-specific CD8+ T-cell epitopes. (A) APCs were infected with vaccinia virus constructs carrying the relevant part of the HCV polyprotein (vHCV 827). After a defined time period (0, 2, 4, 8, 12, 16, 20 and 24 hours) these APCs were added to HCV-specific CTL lines and antigen-specific IFN-γ production was assessed by flow cytometry. (B) Representative flow cytometry plots of a CTL line stimulated with infected APCs. Prior to intracellular IFN-γ staining, the epitope-specific CTL line was stimulated for 5 hours with APCs previously infected with vHCV 827 and pre-incubated for 2, 4, 8 and 12 hours respectively. The background value from the negative control (T7 RNA polymerase (vTF7)) was subtracted from all measured response frequencies (IFN-γ+ CD8+/total CD8+). (C) Representative time course showing intracellular IFN-γ staining of an HLA-A*02-restricted CTL line (filled circles) and an HLA-B*27-restricted (open circles) CTL line stimulated with infected APCs pre-incubated for 0 to 24 hours. The background value from the negative control (vTF7) was subtracted from all measured response frequencies (IFN-γ+ CD8+/total CD8+), which were then normalized to the maximum response by defining the smallest value as 0% and the largest value as 100%. The complete kinetic (0 to 24 hours) was performed for all subjects, and the normalized responses were used to generate the data shown in Figure 8D and E. (D) Responses of CTL lines against APCs infected with vHCV 827 and pre-incubated for 2, 4 and 8 hours. Normalized IFN-γ responses of CTL lines specific for HLA-A*02-restricted epitopes (n = 9, filled circles) and for the immunodominant HLA-B*27-restricted NS5B_2841_ epitope (n = 7, open circles) are shown. Responses were normalized to the maximum response, as described for Figure 8C. Horizontal bars represent mean values. (E) Calculated peptide concentration present at the surface of infected APCs after 2, 4 and 8 hours of incubation with vHCV 827. The concentration of presented peptide restricted by HLA-A*02 (n = 9, filled circles; Table 1) and HLA-B*27 (NS5B_2841_; n = 7, open circles) was calculated by correcting for the individual functional avidity of the respective peptide-specific CTL line. For details of the calculations, see Figure S3. P-values were calculated using the Mann-Whitney U-test. Horizontal bars represent mean values. (F) Representative time course showing intracellular IFN-γ staining of an HLA-B*27-restricted CTL line stimulated with infected APCs incubated for 0 to 8 hours and pre-incubated with (triangles) or without (squares) the proteasome inhibitor epoxomicin. The background value from the negative control (vTF7) was subtracted from all measured response frequencies (IFN-γ+ CD8+/total CD8+). Data are representative of experiments performed with two different HLA-B*27-restricted CTL lines and one HLA-A*02-restricted CTL line.

The rapid proteasomal cleavage of the HLA-B*27-restricted NS5B_2841_ epitope prompted us to investigate whether this leads to faster epitope presentation on the cell surface of APCs. Since it is hypothesized that the timing of antigen abundance at the cell surface of antigen presenting cells during initial priming of naive HCV-specific CD8+ T may influence the immunodominace hierarchy of responding CD8+ T cells [25], [35] and since it is assumed that APCs rather than hepatocytes initially prime naïve HCV-specific CD8+ T cells (reviewed by [36]–[38]), we decided to use subject-derived APCs for our assays. Due to the lack of approved models of expanded subject-derived DCs we decided to use EBV-immortalized B-cell lines that best fit to this model of antigen processing and presentation. HLA-B*27+ and HLA-A*02+ EBV-immortalized B-cell lines were infected with vaccinia virus constructs encoding HCV proteins containing the relevant peptides. Importantly, the sequence of the vaccinia virus construct is identical to the sequence of the polypeptides used for our proteasomal digestion experiments (genotype 1a). Subsequently, after infection the cells were cultured for different incubation times (0–24 h) to allow endogenous antigen processing, then added to peptide-specific CTL lines restricted by HLA-B*27 or HLA-A*02, respectively (**Figure 8A**). Induction of IFN-γ production as a read-out for antigen presentation was measured by intracellular cytokine staining after five hours of cocultivation. As shown for representative subjects in **Figure 8B and C** and for all subjects tested in **Figure 8D**, antigen-specific CD8+ T cell IFN-γ production became detectable within two to four hours of vaccinia virus infection, suggesting that endogenously processed epitopes had already reached the surface of the APC in this time period. Importantly, the induction of IFN-γ in HLA-B*27-restricted CD8+ T cells was higher at two and four hours after infection compared to HLA-A*02-restricted CD8+ T cells, even despite the lower avidity of the HLA-B*27-restricted CD8+ T-cell responses (**Figure 8D**). When corrected for functional avidity in individual subjects (**Figure S3**), a significantly higher level of the HLA-B*27-restricted NS5B_2841_ peptide was presented at the cell surface compared to HLA-A*02-restricted epitopes at these time points (**Figure 8E**). Finally, we analyzed the influence of proteasome inhibitors on the endogenous antigen processing and their effect on the timing of the induction of antigen-specific IFN-γ production of HLA-B*27 and HLA-A*02-restricted CTL lines. By using the proteasome inhibitor epoxomicin we were able to delay the induction of epitope-specific IFN-γ production and considerably decrease the number of responding CD8+ T cells (**Figure 8F**). We also performed control experiments with the proteasome inhibitor lactacystin and saw 30–70% reductions in the number of responding CD8+ T cells (data not shown). These data support the assumption that proteasomal digestion more likely than alternative cytosolic proteases, such as the tricorn, is responsible for the production of HCV-specific epitopes. In sum, these results demonstrate that, following endogenous processing via the proteasome, the HLA-B*27-restricted NS5B_2841_ epitope more rapidly reaches a higher peptide concentration on the cell surface compared to HLA-A*02-restricted epitopes, and that this translates to kinetically enhanced induction of antigen-specific IFN-γ production.

## Discussion

In this study, we set out to determine the immunological mechanisms that contribute to the protective effect associated with the immunodominant HLA-B*27-restricted NS5B_2841_ epitope in HCV infection. Importantly, we found that protection cannot be clearly linked to CD8+ T-cell responses characteristics, such as functional avidity or antiviral efficacy. Indeed, it was the first finding of our study that in chronic infection protective immunodominant HLA-B*27-restricted CD8+ T-cell responses had lower functional avidity than HLA-A*02-restricted CD8+ T-cell responses. These results contrast somewhat with previous studies performed in HIV infection, which have observed that CD8+ T-cell responses restricted by HLA-B alleles have a higher functional avidity than those restricted by HLA-A alleles. Most notably, HLA-B*27-restricted CD8+ T-cell responses specific for the immunodominant HIV Gag-derived epitope KK10, which is associated with slow disease progression, displayed the highest functional avidity when tested using the optimal epitope [15], [16]. In contrast, however, CD8+ T-cell responses towards longer natural processed peptide forms of this epitope have a much lower functional avidity than those against the optimal epitope [29]. Thus, in the infected host CD8+ T-cell cross-recognition of optimal and natural peptide forms may result in biased *in vitro* estimations of functional avidity and magnitude of epitope-specific CD8+ T-cell responses when only using the optimal epitope. In HCV infection, Yerly *et al.* observed higher levels of functional avidity for CD8+ T-cell responses in subjects who cleared the virus compared to CD8+ T-cell responses derived from subjects with chronic HCV infection [19]. Although we also found a higher level of functional avidity for HLA-B*27-restricted CD8+ T-cell responses derived from subjects with resolved versus chronic HCV infection, this functional avidity was still lower compared to HLA-A*02-restricted CD8+ T-cell responses. Collectively, our results suggest that the overall protective effect of HLA-B*27 in HCV infection cannot be linked to superior functional avidity even when we used the optimal HCV-epitope in our experiments. This conclusion is in keeping with a recent study by Harari *et al.*, who did not find a correlation between functional avidity and protective CD8+ T-cell responses specific for several viruses [17].

In addition to functional avidity, we studied other aspects of the CD8+ T-cell response to the protective immunodominant HLA-B*27-restricted NS5B_2841_ epitope. Notably, we found that the antigen-specific naïve CD8+ T-cell precursor frequency for this epitope was not substantially different compared to an immunodominant HLA-A*02-restricted epitope. This is particularly interesting in the light of previous studies suggesting that the number of naïve precursors plays a key role in the generation of immunodominance hierarchies during viral infections in mouse models and humans [21], [23], [24], [39]. However, detailed comparison with subdominant HCV-derived HLA-B*27-restricted epitopes would be required to interpret this observation fully. Furthermore, we found no evidence that CD8+ T-cell responses to the protective immunodominant HLA-B*27-restricted NS5B_2841_ epitope mediated enhanced antiviral efficacy. This observation is somewhat perplexing when searching solely for an immunological explanation for the protective effect of the HLA-B*27-restricted NS5B_2841_ epitope, but gains credence in the context of combined virological considerations. In addition, it should be noted that this parameter was assessed in the context of peptide-pulsed cell lines due to system constraints. In the context of naturally presented epitopes, it remains feasible that rapid and efficient antigen processing could translate into enhanced antiviral efficacy *in vivo*. We finally analyzed the functional profile of HCV-specific CD8+ T cells and found that HLA-B*27- and HLA-A*02-restricted CD8+ T cells have the same combination of functions with similar amounts of produced effector-molecules. However, it is important to note that HLA-B*27-restricted CD8+ T cells have a higher capacity to simultaneously produce multiple effector-molecules compared to HLA-A*02-restricted CD8+ T cells. This is in line with studies performed with HIV-specific CD8+ T cells which have shown that CD8+ T cells specific for the immunodominant HIV KK10-epitope display a superior polyfunctional profile compared to CD8+ T cells restricted by other HLA-alleles [15] and that polyfunctionality is generally connected with a superior control of HIV infection [40]. However, differences in polyfunctionality were much lower in our study compared to these studies performed in HIV infection.

The most important finding of our study is that the protective effect of the immunodominant HLA-B*27-restricted NS5B_2841_ epitope can be linked to extraordinarily rapid processing by both proteasomal forms and fast presentation of the epitope at the cell surface. These important data were obtained by different but complementary, experimental approaches. First, by using biochemical assays with purified constitutive proteasomes and immunoproteasomes, we demonstrated that the region containing the HLA-B*27-restricted NS5B_284_ epitope is rapidly processed resulting in fast generation of epitope precursors. We furthermore show that this rapid processing mainly is due to the very hydrophobic regions flanking the NS5B_284_ epitope, which is in line with the finding of Lucciari-Hartz *et al.* who demonstrated that processing of hydrophobic protein regions of HIV was more efficient than that of hydrophilic regions [41]. In this context, it is important to note that proteasomal digestion generated the optimal HLA-B*27-restricted NS5B_2841_ epitope only in small amounts and mainly produced longer precursors of this epitope. However, these naturally processed longer NS5B_2841_-containing peptide fragments could be cross-recognized by CD8+ T cells derived from chronically HCV infected subjects. These results indicate that HLA-B*27 may be able to present these extended peptide forms [34] and that optimal epitopes are not necessarily the epitope peptide forms produced most frequently in the infected host [29].

Interestingly, the important role of antigen processing and presentation of protective immunodominant viral epitopes has also been demonstrated recently for the protective immunodominant HLA-B*27-restricted HIV Gag-derived KK10 epitope. Specifically, the optimal KK10 epitope was generated only in minimal amounts while epitope precursors of this epitope were produced efficiently by proteasomal cleavage [29]. Therefore, the predominance of proteasomal production of long peptides comprising the NS5B_2841_ epitope, rather than the optimal NS5B_2841_ epitope itself, is similar to the situation observed previously for the KK10 epitope. Likewise, the patterns of CD8+ T-cell cross-recognition and response magnitudes specific for the optimal NS5B_2841_ epitope and the longer NS5B_2841_-containing peptides in our experiments are similar to those found in subjects with chronic HIV infection who respond to the KK10 epitope [29]. However, the HLA-B*27-restricted NS5B_2841_ epitope-containing region is processed significantly faster than the HIV Gag-derived KK10 epitope.

Importantly, by using functional T-cell assays, we could show that the rapid processing of the protective immunodominant HLA-B*27-restricted NS5B_2841_ epitope resulted in early presentation at the surface of APCs. Collectively, these results support the hypothesis that early antigen processing kinetics, rather than absolute epitope quantities, help to elicit the protective immunodominant HLA-B*27-restricted NS5B_2841_ response. This has not previously been observed in humans, but is in agreement with analyses of CD8+ T-cell response hierarchies in normal and immunoproteasome-deficient mice infected with *Listeria monocytogenes*. Indeed, without immunoproteasomal processing, the presentation of an otherwise immunodominant epitope was delayed and failed to evoke a CD8+ T-cell response [25]. Thus, the magnitude and kinetics of antigen-specific CD8+ T-cell responses appear to be determined during the first 24 hours after infection. Furthermore, T-cell response hierarchies were defined before the peak of the inflammatory response and prior to substantial bacterial replication [42]. In addition, competition for the same APC between T cells of different specificities has been shown to occur within the first 5 hours of immunization and to affect the number of T cells responding to a specific antigen in mice [43]. These results may in part be due to the highly regulated capture, processing and presentation of antigens by dendritic cells, which may only have a limited window-of-opportunity to present antigen and engage in long, stable interactions with naïve T cells [35]. Thus, epitopes that are processed and presented faster than others may have a relatively greater chance of evoking early and abundant T-cell responses in infected subjects similar to what has been suggested for infected or immunized mice [25], [35]. This notion is supported by a study showing that the magnitude of HIV-specific CD8+ T-cell responses restricted by HLA-A alleles was dramatically lower in the presence of the protective alleles HLA-B*27 and HLA-B*57, but not in the presence of other HLA-B alleles [44]. In concert with these studies, our results support the biological relevance of rapid processing and presentation in early viral infection, which may in turn lead to the efficient induction of immunodominant and possibly protective CD8+ T-cell responses. Importantly, the analysis of the proteasomal degradation of an artificial chimeric peptide containing of the optimal B27-NS5B_2841_ epitope surrounded by the A2-NS5B_2594_ flanking regions showed that the optimal epitope can be produced in higher amounts in this context compared to that of the natural B27-NS5B_2841_ epitope-containing peptide. These results suggest that for the design of an HCV vaccine construct the timing but also the amount of epitope processed by the proteasome can be manipulated through modifications of the epitope itself as well as of the epitope flanking regions. As discussed above epitope amount may not be as important as the timing of epitope presentation for the priming of virus-specific CD8+ T cells in acute infection; however it may play a more important role for the priming of CD8+ T cells using a vaccine.

Taken together, our findings suggest that immunological factors such as rapid antigen processing and presentation contribute to immunodominance hierarchies and combine with virological factors such as functional constraints on viral escape to generate protective CD8+ T-cell responses in human viral infections such as HIV and HCV. Our results also suggest that HCV immunogens could be modified and optimized *in vitro* to increase the rate of proteasomal processing and thus the likelihood of evoking abundant, or perhaps even immunodominant, CD8+ T-cell responses towards any epitope. This possibility has clear implications for the design of an HCV vaccine.

## Materials and Methods

### Ethics statement

Written informed consent was obtained in all cases and the study was conducted in agreement with the 1975 Declaration of Helsinki, federal guidelines and local ethics committee regulations. The ethics committee of the Albert-Ludwigs-Universität, Freiburg approved the study.

### Study subjects

Twelve subjects with chronic HCV infection (six HLA-A*02+ and six HLA-B*27+) who presented to the University Hospital of Freiburg were included in the study. In addition, six subjects with resolved HCV infection (two HLA-A*02+, one double-positive HLA-A*02+/HLA-B*27+ and three HLA-B*27+) and eleven healthy individuals were included. Peripheral blood mononuclear cells (PBMCs) were isolated from EDTA anticoagulated blood samples using lymphocyte separation medium density gradients (PAA Laboratories GmbH).

### Tetramer enrichment and precursor frequency determination

Procedural details are similar to those described by Alanio et al. [33]. In brief, PBMCs (1–2×10^8^) were incubated for 15 minutes at 4°C with FcR blocking reagent (Miltenyi Biotec) and stained for 30 minutes with pMHCI tetramer conjugated to allophycocyanin (APC), with the pMHCI component at 20 nM final concentration. A small aliquot of the labeled cells was removed for staining (pre-enriched fraction) and the remaining cells were incubated for 20 minutes at 4°C with anti-APC microbeads (Miltenyi Biotec). Again, a small aliquot was removed for counting the pre-enriched fraction and the remaining cells were passed over a magnetic-activated cell separation column (Miltenyi Biotec). After removal of the column from the magnet, the bound cell population (enriched fraction) as well as the flow-through (depleted fraction) were collected and stained. The frequency of the naïve epitope-specific T-cell population was determined using a calculation similar to that of Alanio et al. [33], specifically the absolute number of phenotypically naïve (CD45RA^hi^, CD27^hi^, CCR7^hi^, CD11a^low^) tetramer+ CD8+ T cells/the absolute number of CD8+ T cells was calculated.

### Antibody staining and multiparametric flow cytometry

Cell populations (pre-enriched, enriched and depleted fractions) were labeled with a combination of anti-CD45RA-phycoerythrin (PE), anti-CD27-APC-eFlour780 (eBioscience), anti-CD8-AmCyan, anti-CCR7-PE-Cy7, anti-CD3-Pacific-Blue and anti-CD11a-fluorescein isothiocyanate (FITC) monoclonal antibodies (mAbs; BD Biosciences). Viaprobe (7-AAD; BD Biosciences) was used for the exclusion of dead cells. The cells were stained in PBS supplemented with 5% fetal calf serum (Pan-Biotech) for 20 minutes and washed twice before and after addition of the staining reagents. All samples were acquired using a FACS Canto II flow cytometer (BD Biosciences) and analyzed with FlowJo software (TreeStar Inc.).

### Peptides and tetramers for cellular analyses

Peptides corresponding to immunodominant HCV-epitopes restricted by HLA-A*02 (CINGVCWTV, KLVALGINAV and ALYDVVSKL) and HLA-B*27 (ARMILMTHF), as well as long peptide forms of ARMILMTHF, were synthesized with a free amino and carboxy terminus by standard Fmoc chemistry (Genaxxon Bioscience). All peptides were dissolved and diluted according to previously reported protocols [45]. Tetrameric pMHCI complexes were generated as described previously [46].

### CD8+ selection and polyclonal expansion of PBMCs

Procedural details were performed as described previously [9]. In brief, CD8+ T cells were enriched from PBMCs using magnetic beads coupled to anti-CD8 mAbs (Dynabeads, Dynal) and a particle magnetic concentrator. The enriched CD8+ fractions were cultured in 2 mL complete medium (RPMI 1640 (Gibco) containing 10% fetal calf serum, 1% streptomycin/penicillin (Invitrogen) and 1.5% HEPES buffer, 1 mol/L (Biochrom)) supplemented with 100 U/mL recombinant human interleukin-2 (rIL-2; Hoffmann-La Roche), 0.04 µg/mL anti-human CD3 mAb (Immunotech) and irradiated autologous PBMCs. Twice per week, 1 mL medium supplemented with 200 U/mL IL-2 was exchanged. On day 14, the expanded CD8+ cells were used for intracellular IFN-γ staining.

### Stimulation of PBMCs with synthetic peptides

Experiments were performed as described previously [9]. In brief, PBMCs (4×10^6^) were resuspended in 1 mL complete medium and stimulated with peptide at 10 µg/mL final concentration in the presence of 0.5 µg/mL anti-CD28 mAb (BD PharMingen). On days 3 and 10, 1 mL complete medium supplemented with rIL-2 at 20 U/mL final concentration was added. On day 7, the cultures were restimulated with the corresponding peptide (10 µg/mL) and 1×10^6^ irradiated autologous PBMCs. On day 14, the cells were used for intracellular IFN-γ staining.

### Recombinant expression vectors and kinetic assays for the endogenous processing of peptides

The recombinant vaccinia virus construct vHCV 827, which encodes all relevant HCV peptides according to the sequence of genotype 1a, together with a vaccinia virus encoding the T7 RNA polymerase (vTF7) (both generously provided by Charles Rice, Rockefeller University, New York [47]), were used to induce transient expression of endogenously processed HCV peptides in HLA-matched EBV-immortalized B-cell lines (B-LCLs). B-LCLs were infected at a multiplicity of infection (MOI) of 50 for 1 hour with vHCV 827 and vTF7, or with vTF7 alone as a negative control, and washed. The infected B-LCLs were incubated for defined time periods (0, 2, 4, 8, 12, 16, 20 and 24 hours) at 37°C and added as stimulators to peptide-specific CTL lines prior to intracellular IFN-γ staining. For proteasome inhibition assays B-LCLs were incubated for 1.5 hours with 2 nM epoxomicin (Calbiochem) or 50 nM lactacystin (Calbiochem) prior to vaccinia virus infection.

### Intracellular IFN-γ and multicytokine staining

Procedures were performed as described previously [45]. In brief, peptide-specific CTL lines were stimulated with HLA-matched B-LCLs pulsed with increasing concentrations of corresponding peptide (10^−9^–10^−4^ M), or with HLA-matched B-LCLs infected with vHCV 827, at an E∶T ratio of 1∶1. Cells were then incubated for 5 hours in the presence of 50 U/mL human rIL-2 and 1 µL/mL brefeldin A (BD PharMingen). After incubation, cells were blocked with immunoglobulin G1 (IgG1) and stained with anti-CD8-PE mAb (BD Biosciences). Following fixation/permeabilization with Cytofix/Cytoperm (BD PharMingen), cells were stained with anti-IFN-γ-FITC mAb (BD Biosciences), then fixed in 100 µL CellFIX (BD PharMingen) prior to flow cytometric analysis.

For multicytokine staining cells were incubated for 5 hours in the presence of 10^−5^ M specific peptide, 50 U/mL human rIL-2, 0.325 µL/mL monensin (BD PharMingen) and 0.5 µL/mL brefeldin A (BD PharMingen). After incubation, cells were blocked with IgG1 and stained with anti-CD8-APC H7 (BD PharMingen) mAb. Following fixation/permeabilization with Cytofix/Cytoperm, cells were stained with anti-CD107a-PE (BD PharMingen), anti-IFN-γ-eFlour450 (eBioscience), anti-IL-2-PerCP-Cy5.5 (BioLegend), anti-MIP-1β-FITC (R&D Systems), and anti-TNF-α-PE-Cy7 (BD PharMingen) mAbs, or anti-IFN-γ-eFlour450, anti-IL-4-PE (BD PharMingen) and anti-IL-10-APC (BD PharMingen) mAbs or anti-IFN-γ-eFlour450, anti-IL-17A-PE (eBioscience) and anit-IL-22-APC (R&D Systems) mAbs then fixed in 100 µL CellFIX prior to flow cytometric analysis.

### Cell culture

HuH7-Lunet cells were transduced with the JFH1-based selectable subgenomic luciferase replicon and a selectable lentiviral vector expressing the complementary DNA of HLA-A*02 (HuH7_A2_HCV) or HLA-B*27 (HuH7_B27_HCV) [32]. Generation of the HLA-B*27 expressing vector [48], the HLA-A*02 expressing vector [32] and the JFH1-based selectable subgenomic luciferase replicon [32] were performed as described previously. Cells were grown in Dulbecco's modified Eagle medium high glucose (4.5 g/L) with stable glutamine (PAA Laboratories GmbH) supplemented with 10% fetal calf serum, 1% penicillin/streptomycin and nonessential amino acids (Biochrom). For continuous passage, the culture medium was supplemented with 1 mg/mL G418 (PAA Laboratories GmbH) and 10 µg/mL blasticidin S hydrochloride (Carl Roth GmbH & Co).

### Viral inhibition assay

HuH7_A2_HCV and HuH7_B27_HCV replicon cells were pulsed for 1 hour with increasing concentrations of corresponding peptide (10^−9^–10^−4^ M) and washed intensively. Pulsed replicon cells were then cocultured with peptide-specific CTL lines at an E∶T ratio of 1∶1. After 24 hours, the inhibition of viral replication was measured by luciferase activity.

### Luciferase assay

Luciferase activity was detected using the Steady-Glo Luciferase Assay System (Promega) and measured with Luminoskan Ascent (Thermo Fischer) and expressed as relative luciferase unit (RLU).

### Purification of 20S proteasomes

Constitutive and immuno-20S proteasomes were purified from LCL721.174 and LCL721 human EBV-transformed B cell lines as described previously [49]. LCL721.174 originate from LCL721 but carry only one copy of chromosome 6, which contains a deletion of the TAP1 and 2, LMP2 and LMP7 genes in the MHC class II region.

### Peptide synthesis, HPLC and purification for processing studies

Peptides were synthesized on a MK-IV peptide synthesizer, HPLC purified and verified by LC-MS on an HPLC Shimadzu QP8000-system (Schafer-N). Additional purification to >98% purity was performed prior to proteasomal peptide digestion experiments using a JupiterProteo (250×2.1 mm) column (Phemomenex) on a SMART HPLC system (Amersham) as described previously [29].

### *In vitro* proteasomal peptide digests and mass spectrometry analyses

Proteasomal digestions were performed as described previously [29]. In brief, 2 nmol of each peptide was incubated for 2, 4 and 6 hours with 2 µg (or 4 µg in the degradation experiment) of either purified immunoproteasome or constitutive proteasome in digestion buffer (20 mM HEPES-NaOH pH 7.6, 2 mM MgAc_2_, 0.5 mM DTT), and samples were diluted 1∶5 before injection into the mass spectrometer. All peptide digests were performed on the same day. Although a range of peptide fragment lengths was obtained following digestion of the two HLA-A*02-restricted epitope-containing 25-mers (NS3: ^1065^AT^1099^, A02-CINGVCWTV; and, NS5B: ^2587^VY^2611^, A02-ALYDVVSKL), similar to results reported previously for HIV 25-mer peptides [29], the HLA-B*27 epitope-containing peptide (NS5B: ^2833^ID^2857^, B27-ARMILMTHF) was completely degraded; only 4–9 mer peptides were detected, and none contained the epitope. Consequently, the experiments were repeated with a 1∶4 dilution of the purified immunoproteasome or constitutive proteasome stock solution, and these samples were diluted 1∶2 before injection into the mass spectrometer. Mass spectrometry analyses of peptide digests were performed as described previously [29]. In brief, peptide digests were analyzed by nanoscale liquid chromatography using a Waters NanoAcquity UPLC system, with a Waters NanoEase BEH-C18, 75 µm×15 cm reversed phase column. Mass spectrometry analysis of peptide fragments was performed using a Waters Q-Tof Premier in positive Vmode equipped with a nano-ESI. For fragment identification and relative quantification of the peptide fragments, the instrument was run in MS^E^-mode. Each sample was analyzed in triplicate. Data processing, fragment identification and quantification of LC-MS^E^ data was performed using ProteinLynx Global Server (PLGS) version 2.2 or MassLynx4.1 software. Quantification of the remaining substrate was performed using LC-MS^E^ data and ProteinLynx Global Server (PLGS) version 2.2, and peptide identifications were assigned by database searches containing the full-length peptide sequences. The mass error tolerance values were mostly under 5 ppm. Precursor degradation rates were quantified manually via extracted ion chromatograms.

### Statistical analysis

Statistical analysis was performed using GraphPad Prism 5 software (GraphPad Prism Software, Inc.). P-values were calculated using the Mann-Whitney U-test.

## Supporting Information

Figure S1 **Proteasomal digestion occurs at ^2857^D and C-terminal to ^2857^D.** (A) The relative production of B27-NS5B_2841_-containing digestion products with specific C-terminal endings (F, A, R, D, Q and L) by constitutive proteasomes and immunoproteasomes for 1 to 6 hours using standard conditions. For these experiments a C-terminally extended B27-NS5B_2841_-containing 25-mer peptide was used (amino acid sequence as indicated, the NS5B_2841_ epitope sequence is underlined). Data are representative of triplicate experiments. (B) Sequences of all B27-NS5B_2841_-containing digestion products ending at F, A, R, D, Q and L respectively.
(TIF)

Figure S2 **Production of optimal epitopes.** Production of optimal B27-NS5B_2841_ and A2-NS5B_2594_ epitopes in percentage of all peptide fragments produced following constitutive and immunoproteasomal digestion, respectively, of the artificial chimeric B27-in-A2 peptide and the two natural A2-in-A2 and B27-in-B27 amino acid sequences.
(TIF)

Figure S3 **Calculation of peptide levels present at the surface of infected antigen-presenting cells.** Representative calculation of peptide levels present at the surface of infected APCs after 8 hours of incubation. The normalized frequency of the IFN-γ response that is achieved after a defined time of incubation can be transferred to the peptide titration curve and used to identify the peptide level that is required to achieve the same response frequency.
(TIF)
