# Supplementary figures and images for: Rapid Antigen Processing and Presentation of a Protective and Immunodominant HLA-B*27-restricted Hepatitis C Virus-specific CD8+ T-cell Epitope
Source: PLoS Pathog. 2012 Nov 29;8(11):e1003042. doi: 10.1371/journal.ppat.1003042 (PMC3510254; doi:10.1371/journal.ppat.1003042)

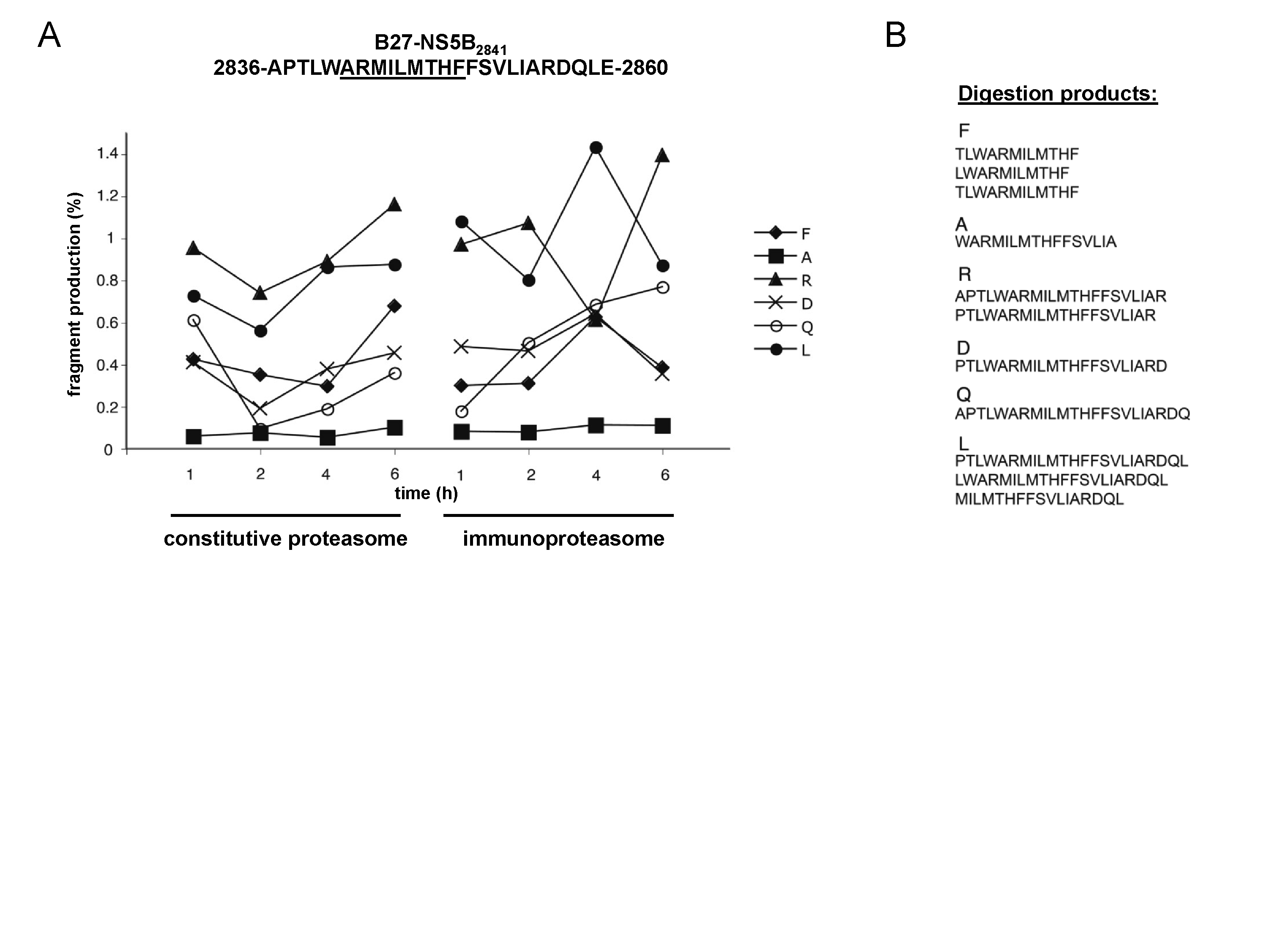

Supplement: Figure S1 — Proteasomal digestion occurs at 2857D and C-terminal to 2857D. (A) The relative production of B27-NS5B2841-containing digestion products with specific C-terminal endings (F, A, R, D, Q and L) by constitutive proteasomes and immunoproteasomes for 1 to 6 hours using standard conditions. For these experiments a C-terminally extended B27-NS5B2841-containing 25-mer peptide was used (amino acid sequence as indicated, the NS5B2841 epitope sequence is underlined). Data are representative of triplicate experiments. (B) Sequences of all B27-NS5B2841-containing digestion products ending at F, A, R, D, Q and L respectively. (TIF) [file ppat.1003042.s001.tif]
